# *NLRP6*-Dependent Pyroptosis-Related lncRNAs Predict the Prognosis of Hepatocellular Carcinoma

**DOI:** 10.3389/fmed.2022.760722

**Published:** 2022-03-02

**Authors:** La Zhang, Xiuzhen Zhang, Wei Zhao, Xinyu Xiao, Shanshan Liu, Qiling Peng, Ning Jiang, Baoyong Zhou

**Affiliations:** ^1^Department of Hepatobiliary Surgery, The First Affiliated Hospital of Chongqing Medical University, Chongqing, China; ^2^School of Basic Medical Science, Chongqing Medical University, Chongqing, China; ^3^Department of Pathology, Chongqing Medical University, Chongqing, China

**Keywords:** hepatocellular carcinoma, lncRNA, NLRP6, pyroptosis, prognostic model

## Abstract

Pyroptosis, a novel pro-inflammatory type of programmed cell death, is involved in the tumorigenesis of various cancers. Recent findings have implicated long non-coding RNAs (lncRNAs) in the serial steps of cancer development. However, the expression and prognostic signatures of pyroptosis-related lncRNAs in hepatocellular carcinoma (HCC) remain largely unknown. Therefore, a pyroptosis-related lncRNA prognostic model was constructed for HCC. Thirty-four pyroptosis-related genes were obtained from previous reviews, and gene expression data were collected from The Cancer Genome Atlas (TCGA) database. Spearman's correlation test was used to identify potential pyroptosis-related lncRNAs. Cox and LASSO regression analyses were used to construct a prognostic model. Subsequently, receiver operating characteristic (ROC) curves were constructed to assess the model's predictive ability for the overall survival (OS) of HCC patients. CytoHubba was used to screen out the potential hub gene, whose expression was verified using clinical samples from HCC patients. Finally, nine pyroptosis-related differentially expressed lncRNAs in HCC were identified, and a prognostic model with four pyroptosis-related lncRNAs was constructed with an area under the ROC curve (AUC) of approximately 0.734. Single-sample gene set enrichment analysis and TCGA revealed different immune infiltration and immune checkpoints between the two risk groups. Moreover, these lncRNAs are closely related to the pyroptosis-related gene, NLRP6, which may be considered a hub gene. NLRP6 was lower-expressed in HCC samples, and patients with lower expression of NLRP6 had the longer OS. In conclusion, NLRP6-dependent pyroptosis-related lncRNAs play important roles in tumor immunity and may be potential predictors and therapeutic targets for HCC.

## Introduction

Hepatocellular carcinoma (HCC), the main type of primary liver cancer, is the second leading cause of cancer-related deaths worldwide (1). The conventional clinical curative options for HCC are surgical resection, tumor ablation, arterial catheter-based treatment, liver transplantation, and recently, targeted therapeutic agents such as sorafenib, which are used for advanced HCC patients. However, the prognosis of patients remains quite bleak, with a 10–12% 5-year survival rate (2, 3). Therefore, there is an urgent need to explore useful prognostic biomarkers and therapeutic targets for patients with HCC.

Long non-coding RNAs (lncRNAs), a subset of non-protein-coding RNA molecules longer than 200 nt, have recently been shown to be involved in the occurrence, development, progression, and metastasis of malignancies (4–6). Growing evidence suggests that lncRNAs in tumors can be regarded as “molecular driver events,” resulting in tumor initiation and progression (7). In addition, a pharmacological study found that the inhibition of certain tumor-specific lncRNAs could inhibit the growth and metastasis of tumors and improve sensitivity to cancer therapy. Pan et al. found that the lncRNA PDPK2P could interact with PDK1 to promote HCC progression via the PDK1/AKT/caspase-3 signaling pathway (8). Ni et al. (9) reported that a novel lncRNA uc.134 may repress HCC progression by regulating post-translational modifications, including phosphorylation and ubiquitination of LATS1 and YAPS127 to inhibit YAP and activate Hippo kinase signaling. It is believed that large-scale screening accompanied with validation experiments could propose highly specific lncRNAs as clinical diagnostic, prognostic, and potential therapeutic biomarkers.

Pyroptosis, a novel form of programmed cell death, was first defined in 2001 as a unique pro-inflammatory death pathway (10, 11). In the pyroptotic process, activation of caspases by inflammasomes (cytosolic supramolecular complexes, also known as pyroptosomes) leads to the maturation and activation of proinflammatory cytokines and pore-forming protein gasdemins (GSDMs). Subsequently, the cell membrane ruptures, and cytokines (such as IL-18 and IL-1β) and various damage-associated molecular pattern (DAMP) molecules are released to recruit more immune cells, further perpetuating the inflammatory cascade and leading to cell death (12). The morphological characteristics of pyroptosis include cellular swelling, appearance of bubble-like protrusions, an intact nucleus, and pore formation, and its mechanism mainly depends on the activation of gasdemin D (GSDMD) (13). Recently, an evidence has demonstrated a pivotal association between pyroptosis and multiple tumors. Gao et al. found that GSDMD was an independent prognostic marker for non-small cell lung cancer (14). Wang et al. discovered that GSDMD markedly suppressed the proliferation of gastric cancer cells *in vivo* and *in vitro* (15). Chun et al. reported that berberine inhibited the viability, migration, and invasion capacity of HepG2 cells through the induction of caspase-1 dependent pyroptosis both *in vitro* and *in vivo* (16). Although pyroptosis has been shown to be involved in the pathogenesis of HCC, the exact mechanisms underlying pyroptosis require further study. Some studies have suggested that lncRNAs regulate pyroptosis in tumors (17, 18), but pyroptosis-related lncRNAs in HCC have rarely been reported.

**Figure 1:**
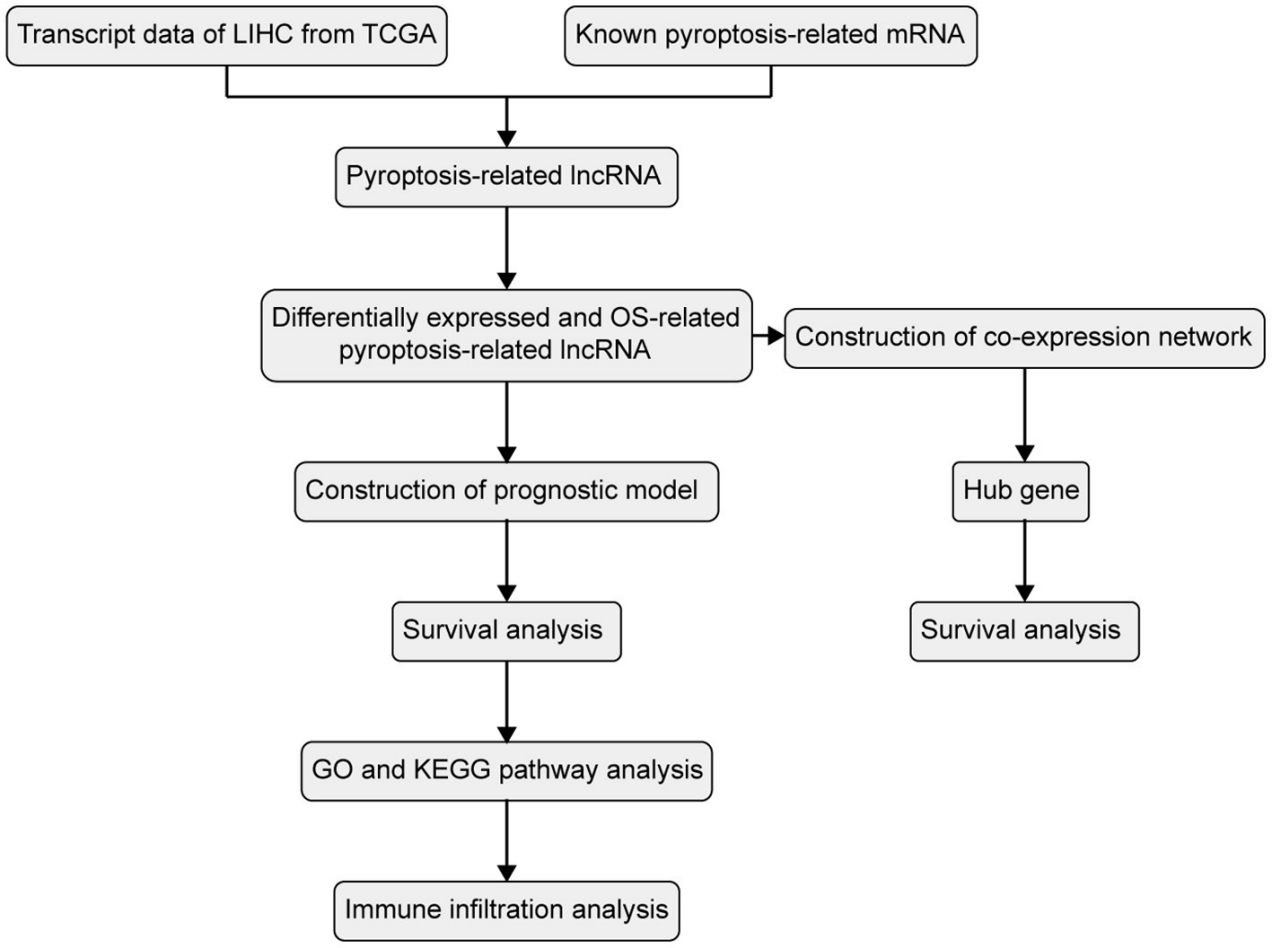
Flowchart of construction and analysis of the prognostic model.

Based on the studies outlined above, as shown in Figure 1, in this study, the intersection between pyroptosis-related genes and differentially expressed lncRNAs in HCC was investigated and pyroptosis-related lncRNAs were identified by systemic bioinformatic analysis based on The Cancer Genome Atlas (TCGA) database. A novel pyroptosis-lncRNA prognostic model was constructed, and its relationship with the tumor immune microenvironment was explored.

## Materials and Methods

### Raw Data Collection and Analysis

RNA-sequence data (50 normal liver tissues and 369 HCC tissues) and clinical data were acquired from the TCGA database (https://portal.gdc.cancer.gov/) and merged using the Practical Extraction and Perl script and the clinical features are detailed in Table 1. Thirty-four pyroptosis-related genes were identified from previous reviews (10, 19–21). Spearman's correlation test was conducted to explore potential pyroptosis-related lncRNAs, and thresholds with |*R*2| > 0.4 and *P* < 0.001 were considered as the correlation coefficients. The raw count data were normalized using the edgeR R package using filtering criteria of adjusted *P* < 0.05, and |log2 (fold change) | > 1 to identify the significant differentially expressed lncRNAs between the normal and tumor tissues. The pyroptosis-related genes, potential pyroptosis-related lncRNAs, and significant differentially expressed lncRNAs are presented in Supplementary Table S1. Subsequently, Cytoscape (version 3.8.0) was used to visualize the co-expression network between pyroptosis-related differentially expressed lncRNAs and pyroptosis-related genes (22).

**Table 1:** Clinical pathological parameters of patients with HCC.

**Feature**	***N* (339)**	**%**
**Age (years)**		
<65	208	61.4
≥ 65	131	38.6
**BMI**		
≤ 24	152	44.8
> 24	162	47.8
Unknown	25	7.4
**Gender**		
Male	231	68.1
Female	108	31.9
**Histologic grade**		
G1	46	13.6
G2	166	49.0
G3	113	33.3
G4	12	3.5
Unknown	2	0.6
**TNM stage**		
I	170	50.1
II	84	24.8
III	81	23.9
IV	4	1.2
**Residual tumor**		
R0	301	88.8
R1	10	2.9
R2	2	0.6
R3	19	5.6
Unknown	7	2.1

### Establishment of a Pyroptosis-Related lncRNA Prognostic Model for HCC

Based on the computational models of previous reports (23, 24), first, a univariate Cox regression analysis was conducted to discover underlying pyroptosis-related lncRNAs associated with overall survival (OS) with a threshold standard of *P* < 0.01. Subsequently, by using the R package “glmnet”, least absolute shrinkage and selection operator (LASSO) Cox regression analysis (with the penalty parameter estimated by 10-fold cross-validation) was conducted to reduce the number of candidate lncRNAs and strengthen their clinical applicability (25, 26). Finally, multivariate Cox regression analysis was used to construct the prognostic signature, with the risk score formula calculated as follows:


$$\begin{array}{l} survival\;risk\;score\;(patient)=\\ \overset{k}{\underset{i=1}{\sum^{\text{​}}}}\text{coefficient (gene i) expression value of (gene i)} \end{array}$$

(where “κ” represents the total number of pyroptosis-related lncRNAs in the prognostic model, “gene i” represents the ith pyroptosis-related lncRNA, and “coefficient (gene i)” represents the coefficient of the pyroptosis-related lncRNA in the multivariate Cox regression analysis.)

Patients were divided into two risk-type subgroups, namely the low- and high-risk score groups, according to the median value of the risk score. In addition, the pyroptosis-related lncRNAs, filtered through the univariate Cox regression analysis, were validated for prognostic correlation by generating Kaplan-Meier survival curves using the GEPIA database (http://gepia.cancer-pku.cn/).

### Predictive Power of the Prognostic Model for HCC

Univariate and multivariate Cox regression analyses (Survival R package) were performed to assess the predictive ability of the risk score calculated using the constructed prognostic model. T-distributed stochastic neighbor embedding (tSNE) was performed using Seurat's implementation of principal component analysis (PCA) and tSNE. Receiver operating characteristic (ROC) curves (survival ROC R package) of the calculated risk scores and clinicopathological factors were generated to evaluate predictive ability. The predictive nomogram was used to integrate the prognostic model for predicting the 1-, 3-, and 5-year OS of patients with HCC.

### Immune Infiltration Analysis

The immune cell components and the immune response in the high-risk group were compared with those in the low-risk group, based on the single-sample gene set enrichment analysis (ssGSEA) shown on a heatmap.

### ssGSEA Based on the Risk Score

Differentially expressed genes (DEGs) between the low- and high-risk groups were filtered from the LIHC-TCGA database, with a threshold of |log2 fold change| (|log2FC|) > 1 and false discovery rate (FDR) < 0.05. Gene ontology (GO) enrichment and KEGG pathway analyses of DEGs were performed to explore the potential biological processes and pathways of the upregulated DEGs in the high-risk group. R packages including tidyverse, edgeR, ggbeeswarm, ggsignif, clusterProfiler, and ggplot2 were used for the data analysis. ssGSEA was used to evaluate immune infiltration between the two groups.

### Deep Learning for the Hub Gene

To explore the potential molecular mechanisms of selected pyroptosis-related lncRNAs, CytoHubba, a Cytoscape plugin, was used to identify the hub gene through the 11 topological analysis methods (including MCC, DMNC, MNC, Degree, EPC, BottleNeck, EcCentricity, Closeness, Radiality, Betweenness, Stress, and ClusteringCoefficient) (27). The co-expression of these lncRNAs with this hub gene was shown in a heatmap drawn using the R package ggplot2. The expression and correlation of prognosis and immune infiltration in HCC were subsequently analyzed in terms of mRNA expression levels using the TCGA database and in terms of protein expression levels based on the immunohistochemistry (IHC) staining data obtained from the Human Protein Atlas (https://www.proteinatlas.org/) and our own experiments. Immune infiltrate levels were determined using TIMER (https://cistrome.shinyapps.io/timer/).

### Immunohistochemistry (IHC) Staining

The study protocol was approved by the Institutional Research Ethics Committee of Chongqing Medical University, and written informed consent was obtained from all patients. HCC and normal tissues were obtained and fixed in formalin. Formalin-fixed tissues were embedded in paraffin wax and cut into 4 μm-thick sections. After deparaffinization and rehydration in a xylene-ethanol series, the paraffin sections were incubated with the NLRP6 primary antibody (1:50; Sangon Biotech; D261937) at 4°C overnight and with secondary antibodies at 37°C for 30 min. After washing, the samples were counterstained with hematoxylin, dehydrated through an ethanol-xylene series, and covered with neutral balsam (Biosharp; BL704A). Staining was assessed and scored as 0, 1+, 2+, and 3+ by a qualified pathologist who was blinded to this study.

### Statistical Analysis

Statistical analysis was conducted using R Studio (version 1.3.1093). Continuous variables such as gene expression levels were analyzed using the single-factor analysis method. For categorical variables, Pearson's chi-square test was used. The Kaplan-Meier method with a two-sided log-rank test was applied to compare the OS of patients in the two groups. Univariate and multivariate Cox regression analyses were employed to evaluate the prognostic value of the risk signature model. The Mann-Whitney test was used to compare immune cell infiltration between the two groups.

## Results

### Construction of a Co-expression Network of Pyroptosis-Related lncRNAs for HCC

**Figure 2:**
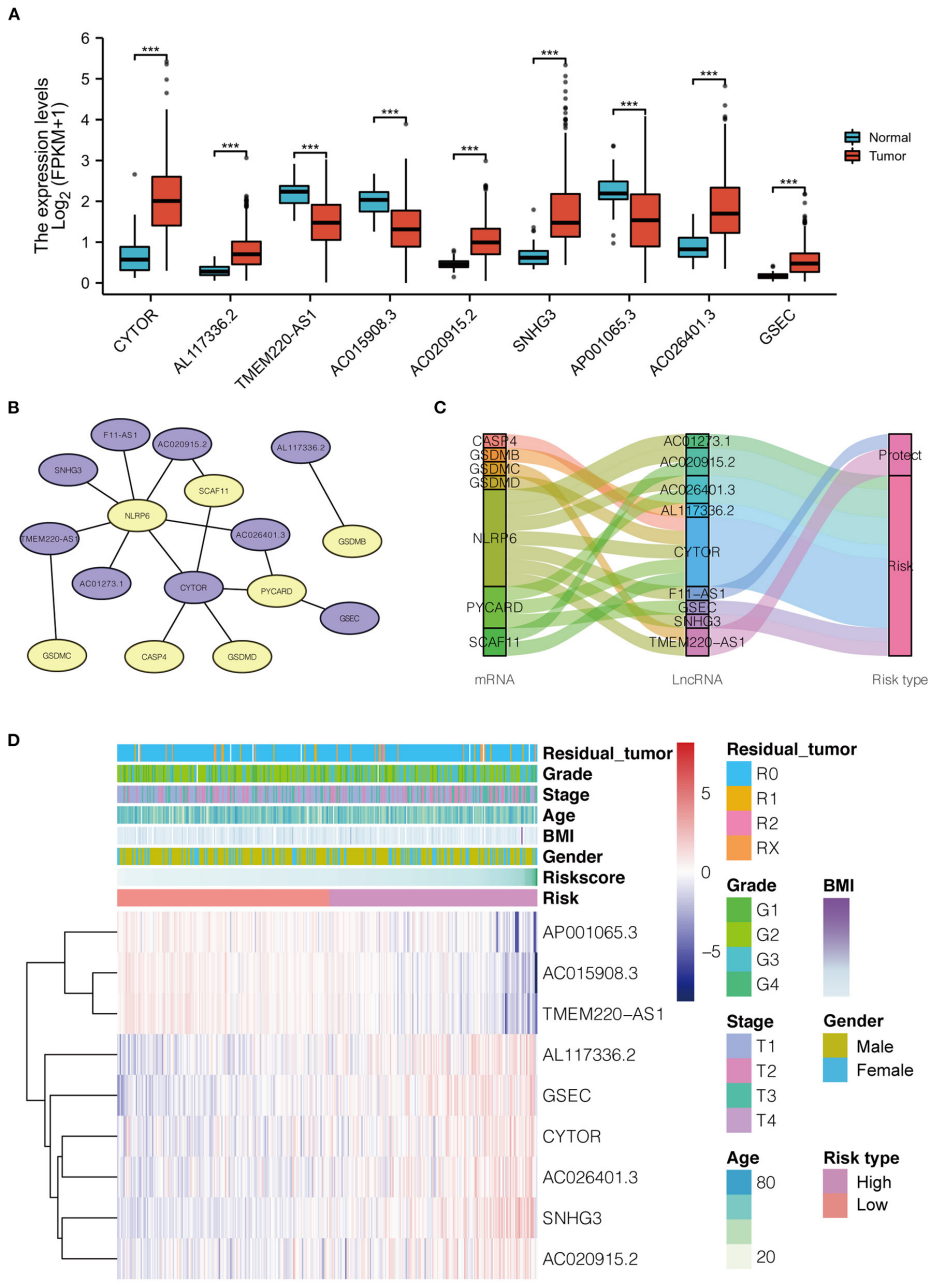
**(A)** The distribution of 9 pyroptosis-related lncRNA expression patterns in tumor and adjacent normal liver tissues based on LIHC-TCGA dataset. **(B)** The co-expression network of the 9 pyroptosis-related lncRNAs-mRNAs with prognostic value was constructed and visualized using Cytoscape (Purple represents lncRNA, and yellow represents mRNA). **(C)** Sankey diagram for pyroptosis-related lncRNAs-mRNAs co-expression network. **(D)** Heatmap for the expressions of 9 lncRNAs and clinicopathologic characters of the low- and high-risk groups. LICH, Liver hepatocellular carcinoma; TCGA, The Cancer Genome Atlas; BMI, Body Mass Index.

Thirty-four pyroptosis-related genes were obtained from authoritative reviews. Furthermore, 908 pyroptosis-related lncRNAs were uncovered from TCGA-HCC cohort data via Spearman's correlation test (|*R*^2^| > 0.4 and *P* ≤ 0.001), of which 140 differentially expressed lncRNAs were selected (with parameters |logFC| > 1, *P* < 0.05) to construct a co-expression network using univariate Cox regression. Ultimately, nine lncRNAs (CYTOR, AL117336.2, AC020915.2, SNHG3, AC026401.3, and GSEC, which were upregulated in HCC, and TMEM220-AS1, AC015908.3, and AP001065.3, which were downregulated in HCC) were selected for further study, as shown in Figure 2A. The co-expression network and the survival correlation of lncRNAs are shown in Figures 2B–D.

### Identification of the Pyroptosis-Related lncRNA Prognostic Signatures and Construction of the Prognostic Model

**Figure 3:**
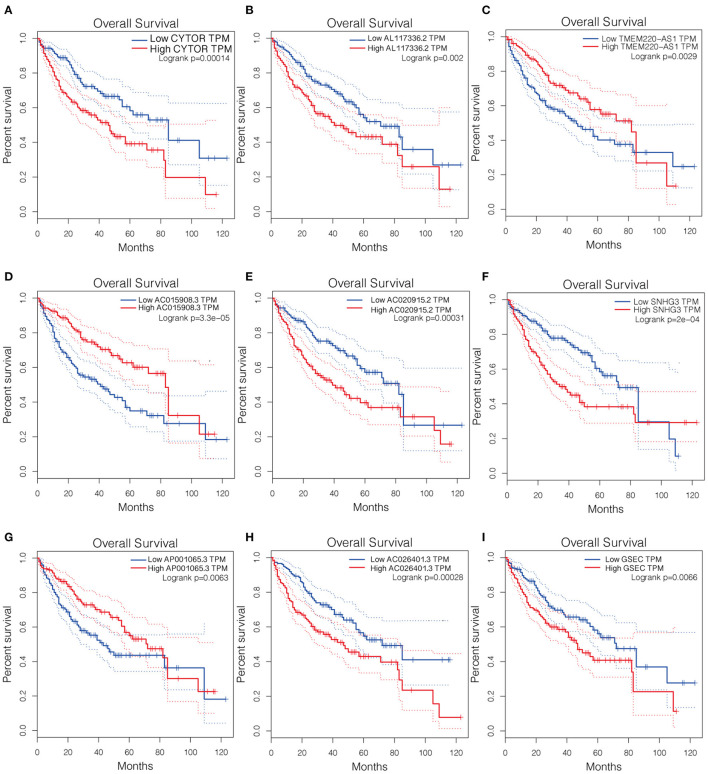
Kaplan-Meier survival curves of the identified 9 pyroptosis-related lncRNAs in LICH-TCGA database using GEPIA. **(A)** CYTOR. **(B)** AL117336.2. **(C)** TMEM220-AS1. **(D)** AC015908.3. **(E)** AC020915.2. **(F)** SNHG3. **(G)** AP001065.3. **(H)** AC026401.3. **(I)** GSEC. LICH, Liver hepatocellular carcinoma; TCGA, The Cancer Genome Atlas; GEPIA, Gene Expression Profiling Interactive Analysis.

**Figure 4:**
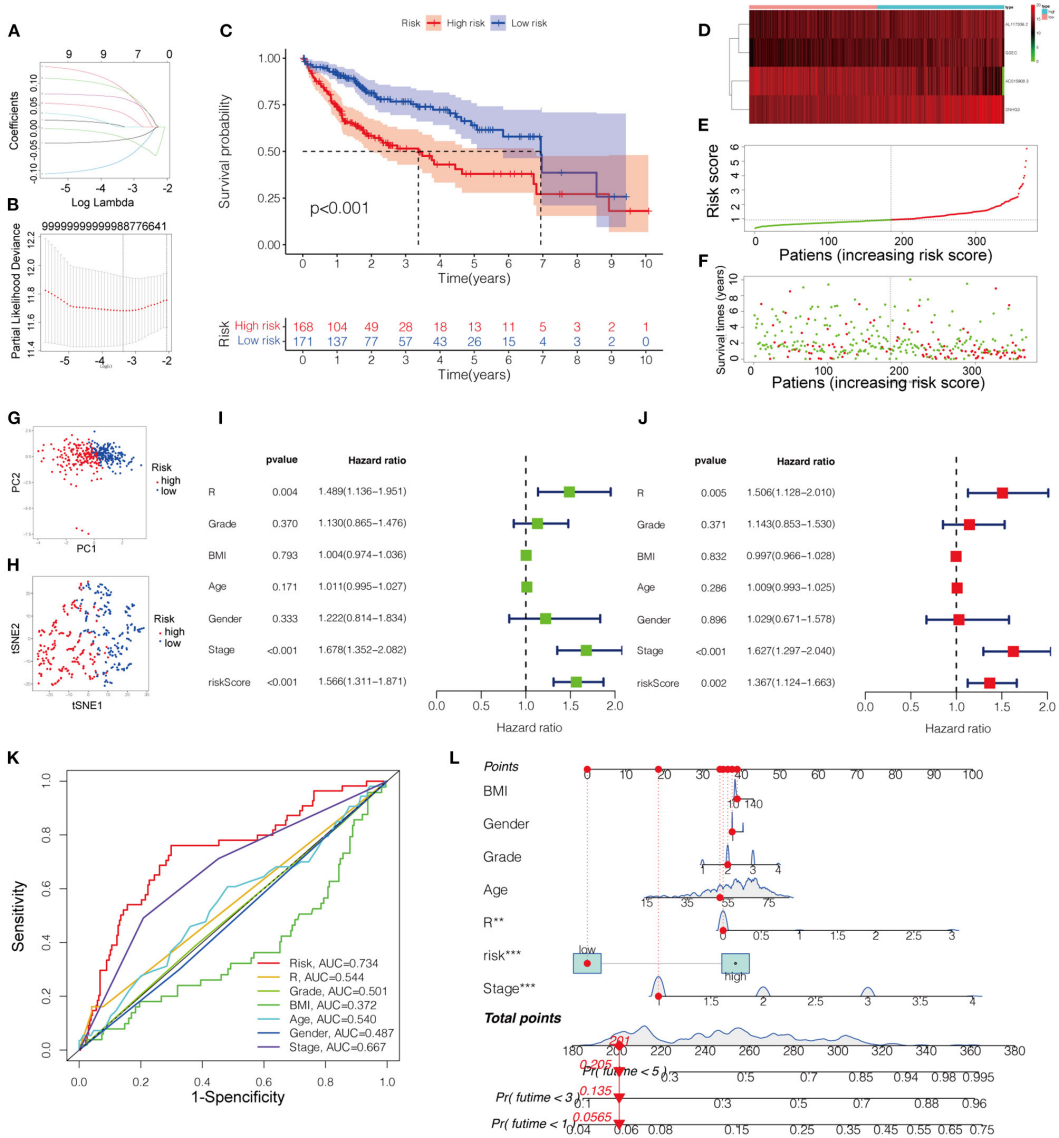
**(A)** LASSO regression of the 9 OS-related genes. **(B)** Cross-validation for tuning the parameter selection in the LASSO regression. **(C)** Kaplan-Meier survival curves for OS of patients in low- and high-risk groups. **(D)** Heatmap for the expression profiles of pyroptosis-related lncRNAs in the low- and high-risk groups. **(E)** The risk curve according to the risk score of each LIHC patient. **(F)** The scatterplot based on the survival status of each patient. **(G)** PCA plot for LICH patient. **(H)** Dot plot for low- and high-risk clusters identified by t-SNE algorithm. **(I,J)** The univariate and multivariate Cox regression analysis of risk score of the prognostic model and clinical features regarding prognostic value. **(K)** The receiver operating characteristic curves (ROC curves) of risk score and clinicopathological characteristics. **(L)** Nomogram for both clinicopathological factors and prognostic pyroptosis-related lncRNAs. OS, overall survival.

The Kaplan-Meier curves of the nine lncRNAs were drawn using GEPIA, as shown in Figure 3. Six upregulated lncRNAs correlated with poor OS were considered potential prognostic biomarkers for HCC patients, while three downregulated lncRNAs (TMEM220-AS1, AC015908.3, and AP001065.3) were correlated with more favorable OS. The number of prognostic model candidates was reduced from nine to eight to achieve the optimum λ value by applying the least absolute shrinkage and LASSO Cox regression analysis (Figures 4A,B). The number was further reduced to four according to the multivariate Cox regression. The final risk score algorithm was as follows:


$$\begin{array}{l} \text{risk score}=(0.1526709^{*}\text{AL1}17336.2\text{ exp.)}\\ \text{​​​​​​​​​​}\;\;\;\;\;\;\;\;\;\;\;\;\;\;-(0.1357352^{*}\text{AC0}15908.3\text{ exp.)}\\ \;\;\;\;\;\;\;\;\;\;\;\;\;\;+(0.1369615^{*}\text{SNHG}3\text{ exp.)}\\ \;\;\;\;\;\;\;\;\;\;\;\;\;\;+(0.1530472^{*}\text{GSEC}\;\text{exp.).} \end{array}$$

Patients were divided into low- and high-risk groups based on the median risk score. The survival status is shown in the Kaplan-Meier curves (Figure 4C). The heatmap revealed differences in the expression of the four pyroptosis-related lncRNAs in low- and high-risk patients in the TCGA dataset (Figure 4D).

### Evaluation of the Prognostic Model

The score of the risk curve and the distribution of the risk score for each patient were then analyzed (Figures 4E,F). Combined with PCA and tSNE projections (Figures 4G,H), the prognostic model had a reliable clustering ability for the risk score in the two cohorts. Patients in the high-risk group had a worse prognosis than those in the low-risk group. Univariate and multivariate Cox regression analyses were performed to evaluate the prognostic value of the constructed model compared with that of the clinicopathological characteristics, revealing that the risk score and disease stage could be independent prognostic factors in HCC (Figures 4I,J).

To further evaluate the predictive sensitivity and specificity of the prognostic model, ROC curves were constructed, indicating the predictability of the prognostic model (Figure 4K). As shown in Figure 4L, the projections from the total points incorporating the prognostic model and clinicopathological characteristics indicated the predicted survival probability at 3 and 5 years for HCC patients.

### Immunity Infiltration Analysis

**Figure 5:**
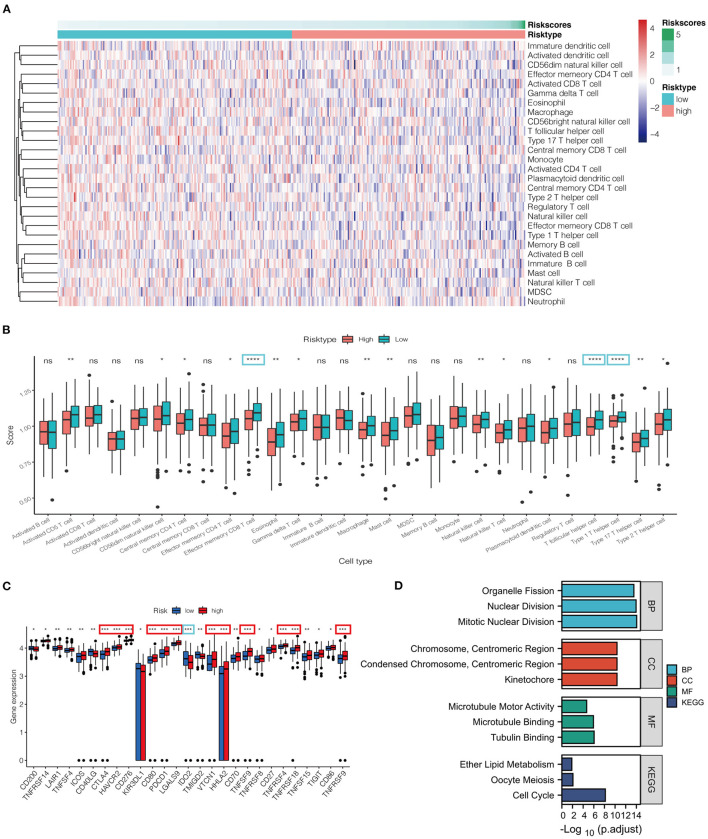
**(A)** Heatmap for immune responses based on ssGSEA algorithms between low- and high-risk groups. **(B)** ssGSEA for the association between immune cell subpopulations and related functions among the two groups. **(C)** Expression of immune checkpoints risk groups between the two risk groups. **(D)** Barplot graph for GO enrichment and KEGG pathway analysis (the more extended bar means the more genes enriched). *P < 0.05, **P < 0.01, ***P < 0.001, ****P < 0.0001, ns, not statistically significant; p. adjust: the *p*-value adjustment; GO, Gene Ontology; KEGG, Kyoto Encyclopedia of Genes and Genomes; BP, biological process; CC, cellular component; MF, molecular function.

Based on ssGSEA, tumor samples classified into low- and high-risk groups were associated with different immune cell infiltrations (Figure 5). The heatmap and box figure indicated that the number of antitumor immune cells, including effect memory CD8 T cells and type 1 T helper cells, was significantly lower in the high-risk group than in the low-risk group (Figures 5A,B). The immune checkpoints were compared between the two groups to explore whether the risk score could guide recommendations for immunotherapy. We found that the expression of CTLA4 and PDCD1 was remarkably increased in the high-risk group compared to that in the low-risk group (Figure 5C).

### Functional Enrichment Analysis

In total, 586 DEGs were identified, of which 248 genes were downregulated and 338 genes were upregulated in the high-risk group (Supplementary Table S2). The particularly enriched network wherein the upregulated DEGs of the high-risk group participated was discovered mainly to be the cell-cycle signaling pathway (Figure 5D).

### Deep Analysis of the Hub Gene

**Figure 6:**
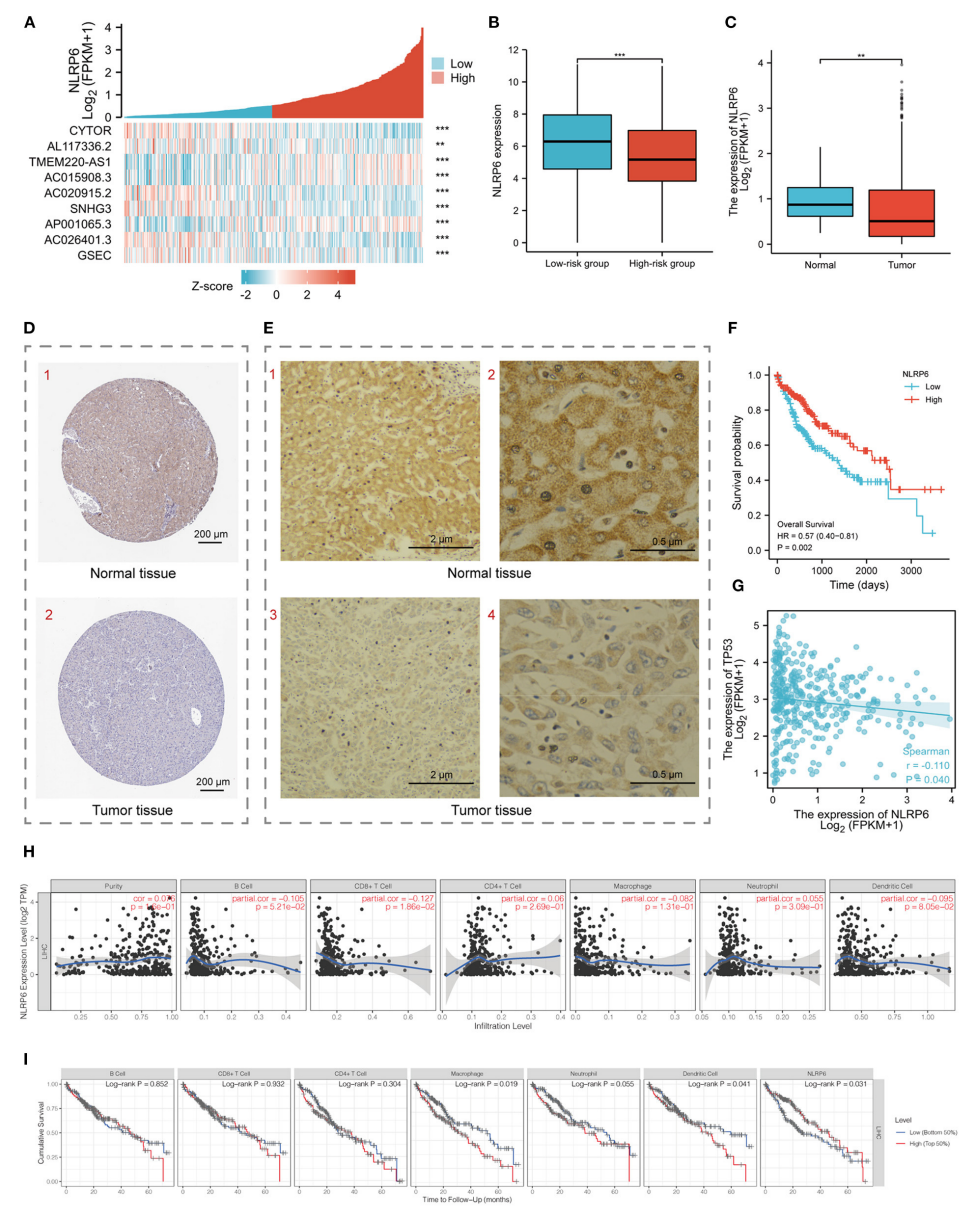
**(A)** Heatmap for the co-expression of 9 pyroptosis-related prognostic lncRNAs with NLRP6. **(B)** Histogram for NLRP6 mRNA different expression between low-and high-risk groups. **(C)** Histogram for NLRP6 different expression between normal and tumor tissues based on LIHC-TCGA database. **(D)** Validation of the expression of NLRP6 on the translational level by the Human Protein Atlas database (immunohistochemistry, IHC), scale bar = 200 μm. **(E)** Representative IHC images for NLRP6 performed on the tissues from one HCC patient. (1, 3) Scale bar = 2 μm, (2, 4) scale bar = 0.5 μm. **(F)** Kaplan-Meier survival curves for the overall survival (OS) based on low/high expression of NLRP6 separated by the median expression. **(G)** Bubble graph for the co-expression of NLRP6 and TP53. **(H)** Relationship between NLRP6 expression and infiltration levels of immune cells in HCC *via* the TIMER database. **(I)** Kaplan-Meier curves revealed the immune infiltration and overall survival rate of HCC. **P < 0.01, ***P < 0.001.

NLRP6 was selected after screening with 11 types of topology analyses (Supplementary Table S3). Meanwhile, the significant co-expression of the nine pyroptosis-related lncRNAs with NLRP6 are shown in the heatmap (Figure 6A). The diminished expression of NLRP6 correlated with the augmented risk score (Figure 6B). The expression of NLRP6 was higher in normal tissues at the mRNA level according to the LIHC-TCGA database (Figure 6C) and at the protein level according to HPA and our experimental results (Figures 6D,E). In addition, patients with higher expression levels of NLRP6 had a higher survival probability (Figure 6F). Moreover, NLRP6 was significantly negatively co-expressed with p53 (TP53), a biomarker of the KEGG cell-cycle signaling pathway (28) (Figure 6G). TIMER was used to demonstrate that the infiltrating levels of CD8 T cells had a notable correlation with NLRP6. Additionally, low levels of macrophages and dendritic cells were related to the poor prognosis of HCC patients with a survival period of <75 months (Figures 6H,I).

## Discussion

HCC, with high prevalence and lethality, is one of the most common cancers worldwide, and its pathological processes remain largely unclear. Furthermore, effective markers to assess disease progression or predict prognosis in HCC are still lacking. Recently, the role of lncRNAs in cancer has been intensively studied, and pyroptosis has been considered to play an important role in cancer development. However, pyroptosis-related lncRNAs in HCC have not yet been reported. In this study, a group of nine pyroptosis-related lncRNAs (CYTOR, AL117336.2, TMEM220-AS1, AC015908.3, AC020915.2, SNHG3, AP001065.3, AC026401.3, GSEC) significantly related to OS were screened to build a prognostic model using univariate Cox regression based on the LIHC-TCGA database. Furthermore, four of nine genes, including AL117336.2, AC015908.3, SNHG3, and GSEC, were identified using LASSO and multivariate Cox regression analysis to construct a more accurate and specific prognostic model. According to this model, the calculated high-risk score suggested a poor OS, indicating that this model would have acceptable ability to predict OS in patients with HCC. Interestingly, our results showed that NLRP6 may be the hub gene for these pyroptosis-related lncRNAs in HCC, and HCC patients with higher expression of NLRP6 may have relatively good prognosis. Moreover, pyroptosis-related lncRNAs may participate in HCC progression by regulating the immune microenvironment of HCC.

LncRNAs have recently been strongly correlated with tumors and may be involved in tumorigenesis, proliferation, migration, and invasion (29, 30). LncRNAs in HCC have become a research hotspot. The most studied lncRNA, HOTAIR, is highly expressed in HCC cells and induces autophagy by regulating autophagy-related 3 (ATG3) and ATG7 (31). Likewise, our study suggests that there are 484 significantly differentially expressed lncRNAs between HCC and normal tissues, of which 140 lncRNAs had co-expression relationships with known pyroptosis-related mRNAs.

Pyroptosis is a novel form of programmed pro-inflammatory cell death, and its effects on tumors have recently attracted much attention. Growing studies have revealed the involvement of lncRNAs in the regulation of pyroptosis, which in turn plays a critical role in many diseases (32). Studies have found that the activation of caspase-1 suppresses the development of HCC by inducing pyroptosis. However, the effects of pyroptosis-related lncRNAs in HCC have not yet been reported. We speculated that there may be a close relationship between pyroptosis-related lncRNAs and HCC. Thus, univariate Cox regression analysis was conducted to identify nine pyroptosis-related lncRNAs associated with the prognosis of HCC patients. Subsequently, LASSO and multivariate Cox regression were used to construct a four-lncRNA prognostic model including AL117336.2, AC015908.3, SNHG3, GSEC, and the AUC of the ROC curve of this model was 0.734, indicating the specificity of the prognostic model.

To further explore the relationship among the pyroptosis-related lncRNAs, 11 algorithms were applied using CytoHubba, and *NLRP6* was identified as the hub gene. Our study shows that all prognosis-related lncRNAs identified here have co-expression relationships with NLRP6. Interestingly, NLRP6 is a sensor protein in the nucleotide-binding domain (NBD) and leucine-rich repeat (LRR)-containing (NLR) inflammasome family, which has recently been shown to regulate pyroptosis by activating caspase-1 (33, 34). This further suggests that lncRNAs included in our prognostic model are associated with pyroptosis. Among our reported lncRNAs, SNHG3 has been demonstrated to be upregulated in HCC and promotes cell proliferation, migration, invasion, and epithelial-mesenchymal transition by regulating cadherin (35), which is considered to be an indicator of pyroptosis (36). These previous findings agree with our bioinformatics analysis results that SNHG3 could be a pyroptosis-related lncRNA involved in HCC. In addition, our results showed that NLRP6 is downregulated in tumor tissues at the transcriptional and translational levels, and patients with higher expression levels of NLRP6 had longer OS. Taken together, we suggest that lncRNAs included in our prognosis model participate in tumor development by regulating the NLRP6-dependent pyroptosis pathway.

Pyroptosis has been identified to be triggered by changes in intracellular and extracellular homeostasis that are associated with innate immunity (37, 38). Furthermore, pyroptosis has been shown to elicit robust anti-tumor immunity both *in vivo* and *in vitro* (39). Wang et al. discovered that pyroptosis increased the sensitivity of 4T1 breast cancer cells to anti-PD1 therapy in a model of athymic nude mice (7). Hou et al. reported that PD-L1 protected cancer cells from host immune T cell-mediated antigen-dependent responses by regulating pyroptosis in a model of breast cancer (40). Additionally, Hage et al. discovered that sorafenib could effectively kill tumor cells by inducing macrophage pyroptosis (41). In our study, the levels of pivotal anti-tumor immune cells were lower in the high-risk group than in the low-risk group, indicating that the former was in an immunosuppressed state. In addition, our study revealed that PD-1 was upregulated in the high-risk group according to the immune checkpoint analysis, which has implications for therapeutic strategies including anti-PD1 treatment.

## Conclusion

Pyroptosis is a novel form of cell death that has attracted much attention for its association with tumor development and tumor resistance. Under these circumstances, several studies have been conducted on pyroptosis; however, few studies have focused on pyroptosis-related lncRNAs in HCC. This study explored several pyroptosis-related lncRNAs, which may regulate pyroptosis through NLRP6, as biomarkers for predicting the survival outcomes of HCC patients. The close relationship between the immune infiltration of HCC and these NLRP6-dependent pyroptosis-related lncRNAs along with NLRP6 provides potential immunotherapy targets for HCC. Given that our pyroptosis-related genes were extracted from previous reviews, the comprehensiveness of the gene inclusion criteria could not be fully guaranteed. Moreover, the lack of experimental verification is a limitation of this study. In conclusion, these NLRP6 dependent pyroptosis-related lncRNAs might provide new insights into the mechanisms underlying tumorigenesis and anti-tumor immunity.

## Data Availability Statement

The original contributions presented in the study are included in the article/Supplementary Material, further inquiries can be directed to the corresponding authors.

## Ethics Statement

The studies involving human participants were reviewed and approved by Institutional Research Ethics Committee of Chongqing Medical University. The patients/participants provided their written informed consent to participate in this study.

## Author Contributions

LZ and NJ conceived and designed this study. LZ and XZ collected and analyzed the relative data. SL collected the clinical samples. XX performed the IHC experiments. LZ wrote the paper. QP and BZ revised the manuscript. All authors read and approved the final manuscript.

## Conflict of Interest

The authors declare that the research was conducted in the absence of any commercial or financial relationships that could be construed as a potential conflict of interest.

## Publisher's Note

All claims expressed in this article are solely those of the authors and do not necessarily represent those of their affiliated organizations, or those of the publisher, the editors and the reviewers. Any product that may be evaluated in this article, or claim that may be made by its manufacturer, is not guaranteed or endorsed by the publisher.
